# Presence of KIR2DL2/S2, KIR2DL5, and KIR3DL1 Molecules in Liver Transplant Recipients with Alcoholic Cirrhosis Could Be Implicated in Death by Graft Failure

**DOI:** 10.3390/diagnostics13071217

**Published:** 2023-03-23

**Authors:** Raquel Morales, José Miguel Bolarín, Manuel Muro, Isabel Legaz

**Affiliations:** 1 Department of Legal and Forensic Medicine, Biomedical Research Institute (IMIB), Regional Campus of International Excellence “Campus Mare Nostrum”, Faculty of Medicine, University of Murcia, 30100 Murcia, Spain; 2 Immunology Service, Instituto Murciano de Investigación Biosanitaria (IMIB), Hospital Clínico Universitario Virgen de la Arrixaca (HCUVA), 30120 Murcia, Spain

**Keywords:** alcoholic cirrhosis, causes of death, KIR genes, sepsis, liver transplant

## Abstract

**Background**: The second-most frequent diagnosis among patients receiving liver transplants (LTs) is alcoholic liver disease. The multifactorial pathophysiology of alcoholic liver disease depends on the innate immune system and the inflammatory cascade. According to recent studies on these receptors, killer-cell immunoglobulin-like receptors (KIRs) may be involved in sepsis, liver rejection, and virus relapse. We aimed to investigate the impact of preclinical issues like ascites and encephalopathy and KIR genetic traits on death from sepsis, multiorgan failure (MF), and graft failure (GF) in AC patients undergoing LTs. **Methods**: We retrospectively reviewed 164 consecutive and deceased Caucasian AC patients who underwent LTs. Pre-transplant complications, cause of death, and patient survival were analyzed. Genomic DNA was taken from peripheral blood, and PCR-SSO was used for genotyping KIR. **Results**: Compared to GF patients, there was a statistically significant increase in the frequency of KIR2DL2+ (75.8% vs. 51.2%; *p* = 0.047). Another increase in frequency was also observed in *KIR2DS2+* in sepsis compared to the GF group (51.2% vs. 43.7%; *p* = 0.018). In patients who passed away from MF, a decrease in *KIR2DL5+* was observed in AC patients with and without encephalopathy (*p* = 0.018). The frequency of *KIR3DL1+* in the AC patients significantly increased the mortality from sepsis (*p* = 0.045), which was confirmed by multivariate logistic regression. The frequency of *KIR3DL1+* in the AC patients significantly increased the mortality from sepsis (*p* = 0.012) and was confirmed by multivariate logistic regression. *KIR2DS1+* and *KIR2DS4+* showed increased mortality due to GF compared to patients without these genes (*p* = 0.011 and 0.012, respectively). However, this fact was confirmed only for *KIR2DS1+* by multivariate logistic Cox regression. **Conclusions**: The presence of the *KIR2DL2/S2+, KIR2DL5+,* and *KIR3DL1+* genes increases the frequency of death from multiple organ failure or graft failure. Our findings highlight the AC patient’s vulnerability to a LT during hospitalization. Following the transplant and outside of it, we adopt essential preventive measures to create a routine healthcare screening to enhance and modify treatments to increase survival.

## 1. Introduction

The second-most frequent diagnosis among patients receiving liver transplants (LTs) is alcoholic liver disease [1,2,3]. Since transplanted AC patients now have a higher survival rate, this liver condition is one of the best indications for LTs [2,4,5]. Despite this improvement, patients should have a thorough post-transplant risk assessment for rejection, virus recurrence, and alcoholic relapse to make extreme efforts to avoid patient death [1,6,7].

Much research has not been performed on the clinical and molecular mechanisms that lead to liver graft loss or death in AC patients, but it has been widely accepted that various factors can contribute to these outcomes [2,8,9,10]. Immune causes of transplant malfunction and death are classically known [11,12,13]. Immune cells are critical for liver graft rejection and patient survival [11,14,15,16,17]. The role of the innate immune system and the inflammatory cascade in the multifactorial pathophysiology of alcoholic liver disease (ALD) is crucial [14,15,18,19,20,21]. Natural killer (NK) cells are thought to be important in the pathogenesis of liver inflammation and injury, as well as the control of viral hepatitis, liver fibrosis, and liver tumorigenesis, according to an increasing number of studies from the past ten years [22,23,24]. A better understanding of the pathogenesis of liver disease has revealed new therapeutic targets for treating this condition, thanks to the characterization of NK cell functions [25]. NK cell immune responses are regulated by a balance of activating and inhibitory signals transmitted by cell surface receptors [26]. According to recent studies, KIRs may be involved in sepsis [27,28], liver rejection [2], and virus relapse [10].

Different studies analyze the relationship between NK cells and their KIRs and find a higher incidence of certain forms of cancer and infections in chronic alcoholics [20,29,30]. 

A delicate balancing act regulates NK cell function between specific interactions between membrane receptors, including KIRs and their ligands, and human leukocyte antigen (HLA) class I molecules [31]. It is still unknown what leads to the deaths and survival of AC patients receiving LTs.

We aimed to investigate the influence of preclinical complications such as ascites and encephalopathy and KIR genetic characteristics on death due to sepsis, multiorgan failure (MF), and graft failure (GF) in patients with CA undergoing LTs to develop preventive actions and improve and/or modify treatments to increase the survival of the transplanted patient.

## 2. Material and Methods

### 2.1. Enrollment of Patients

The University Clinic Hospital “Virgen de la Arrixaca” in the Murcia Region (Spain) performed LTs on 164 consecutive deceased Caucasian AC patients. The medical records of these patients were retrospectively reviewed.

The mean pre-transplant age for all patients analyzed was 54.3 ± 6.9 years (mean years, standard deviation, SD). The inclusion criteria included primary LTs without a history of additional organ transplantation, ABO compatibility, HIV neglect, retransplants, and transplants carried out in patients who lost grafts within the first week following the transplant.

Informed consent was obtained in all cases. The institutional ethical committee approved the study protocol in accordance with the Declaration of Helsinki’s Principles. This study was carried out in accordance with the Helsinki Declaration, and the HCUVA Ethics Committee approved the protocol (PI15/01370). Current regulations ensure the confidentiality of personal data and automated processing.

### 2.2. Alcoholic Cirrhosis Diagnostic Criteria

Clinical, radiologic, and biochemical markers were used to diagnose AC [32]. Before being diagnosed with cirrhosis, AC patients had a history of drinking alcohol above what was considered safe. The opinions of their relatives were considered in the case of a negative self-report of alcohol intake. 

The diagnosis was made after a routine scan, ultrasound test, or clinical examination because the early stages of cirrhosis typically have no symptoms. When symptoms like ascites, upper gastrointestinal hemorrhage, and encephalopathy started to appear during the second stage of decompensated cirrhosis, the illness was, in some cases, not discovered until then. Specific analytical and imaging methods were used to confirm cases of probable cirrhosis.

Additionally, pretransplant ascites and encephalopathy were identified [9,33,34], and it was noted in the clinical history whether they were present.

### 2.3. Diagnosis of Immune Suppression and Rejection

With tacrolimus or cyclosporine as the initial dose, standard triple-drug therapy was administered to all patients, adjusted to maintain the recommended immunosuppressive levels. The Banff scheme and clinical, biochemical, and histological criteria were used to diagnose and grade rejection [33,34]. Liver rejection episodes were treated as previously reported [9,11].

### 2.4. Causes of Mortality in Alcoholic Cirrhosis Patients

The main causes of mortality, MF, GF, and sepsis were analyzed in AC patients according to previous studies [35] until three years after their LTs. Medico-legal autopsies or medical death certificates were used to determine the critical causes of AC death. The principal causes of death are those reported by the physician engaged in the patient’s care.

Other causes of death (cardiac arrest, digestive bleeding, metastasis, edema lung, metastasis, pancreatitis, pneumonia, primary dysfunction, and viral relapse) could not be analyzed individually in this study due to its limited sample size, which does not allow a deeper statistical analysis of the association.

### 2.5. KIR Genotyping

Peripheral blood samples were used in this study. Following the manufacturer’s instructions, the genomic DNA was extracted using the QIAamp DNA Blood Mini Kit from QIAGEN in Hilden, Germany.

Luminex^®^ technology’s sequence-specific oligonucleotides (PCR-SSO) were used for genotyping KIR in all patients (*n* = 164) (TepnelLifecodes, Stamford, CT, USA). This technology activates the *KIR2DS1-S5* and *KIR3DS1* genes by recognizing inhibitory *KIR2DL1-3, KIR3DL1-3,* and *KIR2DL4.* Additionally, we discovered the pseudogenes *KIR2DP1* and KIR3DP1. Activating KIRs (aKIR) have a short cytoplasmic tail and no ITIM motif, whereas inhibitory KIRs (iKIR) have a long cytoplasmic tail and at least one immunoreceptor tyrosine-based inhibitory motif (ITIM) [1,2]. There is one *KIR2DL4* gene, six iKIR (*KIR3DL1-3, KIR2DL1-3*) genes, six aKIR (*KIR3DS1* and *KIR2DS1-5*) genes, and six aKIR (*KIR3DS1* and *KIR2DS1-5*) genes.

### 2.6. Statistical Analyses

The findings and demographic data were entered into a database using SPSS version 27.0 software from Microsoft Corporation, Seattle, Washington (SPSS Software Inc., Chicago, IL, USA). All data are shown as a percentage or mean with a standard deviation. Categorized variables were compared between groups using the 2-tailed Fisher’s exact test and Pearson’s chi-squared test. Long-term AC patient survival differences were evaluated using the Kaplan–Meier and Log-rank tests at three years. A *p* > 0.05 was chosen as the statistically significant level. To assess relative risk, odds ratios (ORs) and 95% confidence intervals (CIs) were created. The factors connected to different death and survival causes were investigated using multivariate logistic regression analysis and multivariate Cox regression methods.

## 3. Results

### 3.1. Clinical and Sociodemographic Characteristics

Table 1 displays the entire cohort’s sociodemographic and primary clinical characteristics (*n* = 164; 91.4% men). Before the LTs, the entire cohort’s average age was 54.3 ± 6.9 (mean years and SD). Four categories were used to group the primary causes of death (sepsis, GF, MF, and others). Cardiovascular collapse, gastrointestinal bleeding, lung edema, metastasis, pancreatitis, pneumonia, primary dysfunction, and viral relapse are additional causes of death.

### 3.2. Frequency of KIR Genes in AC Patients with Different Causes of Death

The frequency of inhibitory and activator KIR genes (iKIR and aKIR, respectively) was examined in patients with various causes of death, as shown in Table 2.

In patients with MF compared to GF, there was a statistically significant increase in the frequency of *KIR2DL2+* (75.8% vs. 43.7%; *p* = 0.018; OR = 0.247; 95% CI: 0.082–0.744). The analysis of the frequency of *KIR2DS2+* revealed an increase that is statistically significant in MF compared to patients who died from GF (75.8% vs. 51.2%; *p* = 0.047, OR = 2.986; 95% CI: 1.037–8.594). On the other hand, an increase was also observed in KIR2DS2+ in sepsis compared to GF (51.2% vs. 43.7%; *p* = 0.018, OR = 4.041; 95% CI: 1.344–12.146).

### 3.3. Analysis of the Frequency of the Presence of KIR Genes in Causes of Death in Patients with and without Ascites

**Figure 1:**
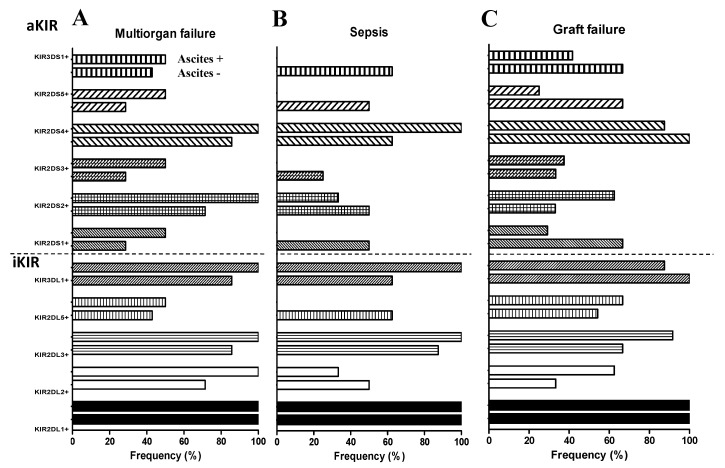
Analysis of the frequency of KIR genes in causes of death in patients with and without ascites. (**A**) Deceased patients due to multiorgan failure. (**B**) Deceased patients due to sepsis. (**C**) Deceased patients due to GF.

No statistically significant differences were found between the three causes of death in analyzing the frequency of ascites and the various KIR genes (Figure 1).

### 3.4. Analysis of the Frequency of KIR Genes in the Causes of Death of AC Patients with and without Encephalopathy

**Figure 2:**
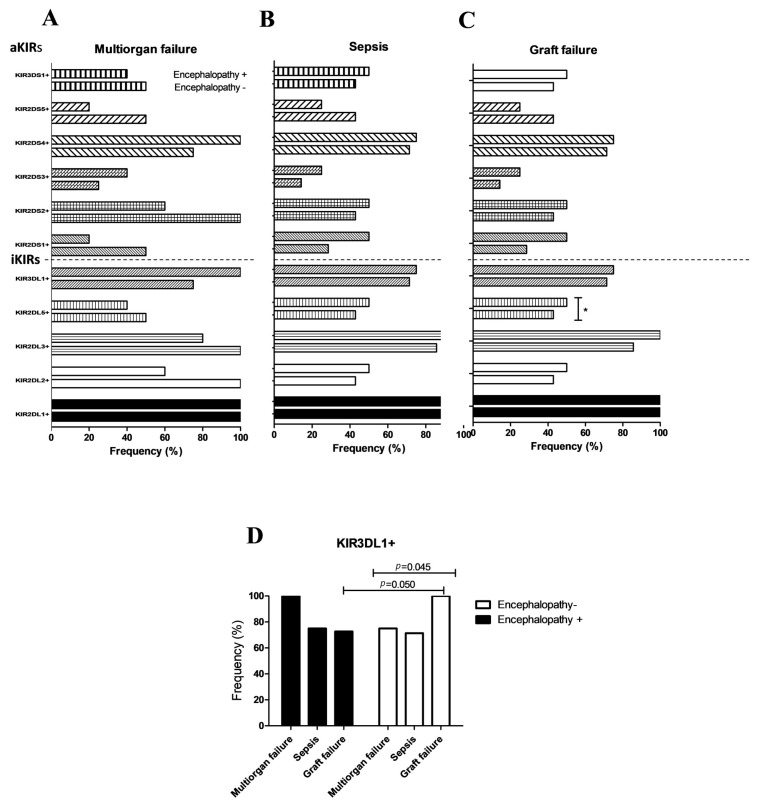
Analysis of the frequency of KIR genes in the main causes of death in patients with and without encephalopathy. (**A**) Deceased patients due to multiorgan failure. (**B**) Deceased patients due to sepsis. (**C**) Deceased patients due to GF. * *p* = 0.018; OR = 2.124; 95% CI: 1.264–2.146, *p* value obtained by comparing encephalopathy-/*KIR2D2DL5+* with encephalopathy+/*KIR2D2DL5+* AC patients. (**D**) Comparison of the different causes of death in AC patients *KIR3DL1+* with and without encephalopathy.

Figure 2 depicts an analysis of the frequency of KIR genes in causes of death in AC patients with and without encephalopathy.

Except for *KIR2DS4+, KIR2DS3+,* and the inhibitor gene *KIR2DL1+*, which were found to be increased in patients with encephalopathy, it was found that the frequency of most KIR genes (activators and inhibitors) decreased in patients with encephalopathy who died from MF (Figure 2A). In the cases of death due to sepsis in AC patients with encephalopathy, the frequency of most of the genes analyzed was increased, except for *KIR2DS5+* (Figure 2B).

The analysis of the frequency of KIR genes in AC patients who died from GF with or without encephalopathy showed an increase in the frequency of most KIR genes (Figure 2B). We only obtained statistically significant differences in the case of the inhibitor gene *KIR2DL5+*. This finding was more representative of AC patients with encephalopathy who died by GF than those without encephalopathy (50% vs. 40.5 %*, p* = 0.018; OR = 2.124; 95% (IC: 1.264–2.146) who died both by GF. This increase in the frequency of *KIR2DL5+* in encephalopathy patients could also be observed in patients who died from sepsis, but no significant differences were obtained in this case. However, when MF patients were compared to AC patients with encephalopathy and patients without encephalopathy, a decrease in *KIR2DL5+* was found in these AC patients.

We also compared the various causes of death to the frequencies of the KIR genes. Only the inhibitor gene *KIR3DL1+* showed statistically significant differences and was significantly higher in AC patients without encephalopathy or death from GF than sepsis or MF (97%, 68%, and 74%, *p* = 0.045). When comparing GF between AC patients with and without encephalopathy, differences in the frequency of *KIR3DL1+* were also discovered (97% vs. 71%, *p* = 0.050; Figure 2D).

This interesting association was confirmed through multivariable logistic regression analysis (*p* = 0.044; Table 3).

### 3.5. Influence of iKIR on Time Elapsed between the Transplantation and the Cause of Death

**Figure 3:**
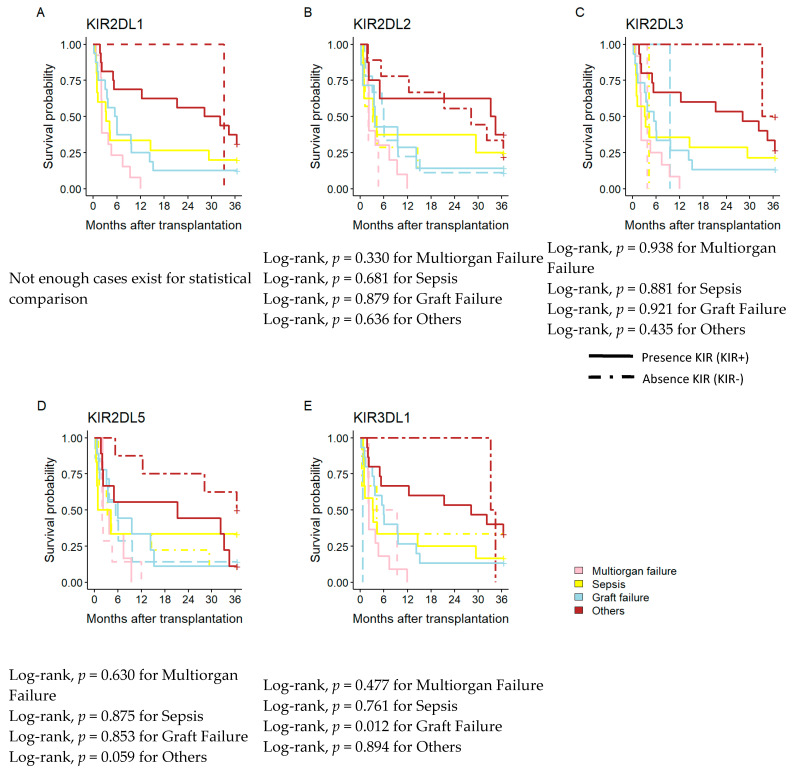
Kaplan–Meier patient survival curves according to the main cause of death and KIR genes. (**A**–**E**) Kaplan–Meier patient survival curves according to the frequency of iKIRs (*KIR2DL1+*, *KIR2DL2+, KIR2DL3+, KIR2DL5+, and KIR3DL1+*). iKIR, inhibitory KIR; aKIR, activatory KIR. Other causes of deaths include; Lung edema, pharynx neoplasia, pancreatitis, pneumonia, chronic rejection, primary dysfunction, and HCV relapse.

Analysis was also performed on the impact of iKIR frequency on AC patient survival over the short- and long-term with various causes of death, including MF, sepsis, GF, and others (Figure 3). There was no statistically significant difference in the frequency of iKIRs (*KIR2DL2, KIR2DL3, KIR2DL5,* and *KIR3DL1*) for either the short- or long-term, nor for the examined causes of death (Figure 3A–D). No patients lacking the *KIR2DL1* gene were present in the population being studied, making it impossible to analyze the influence of this gene. Differences in mortality were found by analyzing *KIR3DL1* at three years (Figure 3E). The frequency of *KIR3DL1+* in the AC patients significantly increased the mortality from graft failure (Log-rank, *p* = 0.012), and this fact was confirmed by multivariate logistic regression.

### 3.6. Influence of aKIR on Time Elapsed between Transplantation and Cause of Death

**Figure 4:**
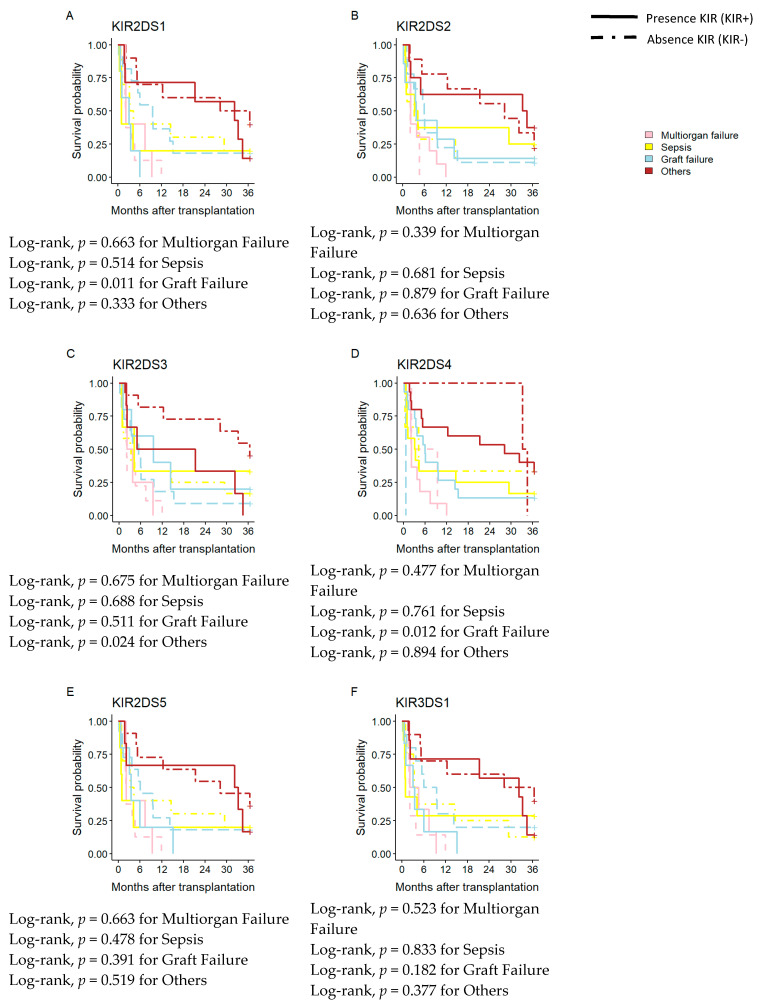
Kaplan–Meier patient survival curves based on the main cause of death and KIR genes. (**A**) Kaplan–Meier patient survival curves according to the presence of KIR2DL3+. (**B**–**F**) Kaplan–Meier patient survival curves according to the presence of *KIR2DS2+*, *KIR2DS3+*, *KIR2DS4+* and *KIR2DS5+*, *KIR3DS1+*.

Additionally, the impact of aKIR frequency on AC patient survival over the short- and long-term with various causes of death (MF, sepsis, GF) was studied (Figure 4).

At three years post-transplant, the analysis of the frequency of aKIRs (*KIR2DS2, KIR2DS5,* and *KIR3DS1*) did not show any statistically significant difference for the causes of death analyzed (Figure 4B,E,F). *KIR2DS1+* and *KIR2DS4+* showed increased mortality due to GF compared to patients without these genes (Log-rank, *p* = 0.011 and 0.012, respectively). However, this fact was confirmed only for *KIR2DS1+* by multivariate logistic Cox regression (Table 4). On the other hand, the *KIR2DS3* analysis showed differences in mortality in patients who died due to other causes of death (Log-rank, *p* = 0.024) (Figure 4C).

## 4. Discussion

In this study, firstly, we characterized our patients by recording their main causes of death, their age, sex, and pre-transplant complications. Subsequently, we analyzed the frequency of the KIR genes in the different causes of death in the total population. Then, we performed the same analysis but divided the population according to whether or not they had a pre-transplant complication (ascites or encephalopathy) to see if they contributed to the KIR genes that cause death. Finally, we analyzed the possible influence of the KIR genes on the period elapsing between when the transplant is performed and when the patient dies. All of this is necessary for preventative actions that build a routine healthcare screening program to improve and modify treatments.

Previous studies [11,36] have observed that KIR genes are involved in rejecting transplanted organs. The KIR genes are a family of immunoreceptors found on the surface of NK cells, a type of white blood cell that plays an essential role in the immune system. These receptors recognize and bind to foreign antigens and initiate an immune response. KIR genes play an essential role in liver transplantation and are associated with an increased or decreased risk of acute liver allograft rejection modulated by NK cell activity.

The results obtained in this study are novel because, for the first time, the KIR genes present in the patients with AC have been related to their causes of death. A study by [35] analyzed the main causes of death without analyzing the patient’s genetics and their influence on the causes of death. Another study by [30] examined how the KIR genes affected the progression of the cirrhotic process in alcoholic patients and found that *KIR2DL2* and *KIR2DS5* had the opposite effect in patients older than 54 years with AC. In these patients, the presence of *KIR2DL2* seemed to protect against AC, whereas the presence of *KIR2DS5* seemed to promote the fibrotic process, especially in those without an associated viral infection. However, in most studies, the KIR genes are analyzed by focusing mainly on organ transplantation since these molecules are the membrane receptors of the NK cells of the innate immune system, whose ligands are the HLA-C molecules that are directly involved in the tolerance or rejection of grafted organs [11,36].

Our data show that most of the patients included in this study are male because women are underrepresented in the final stages of this disease, leading to loss of the liver and a liver transplant [1]. Data previously published by our group showed that alcoholic cirrhosis is a liver disease closely associated with men due to their high alcohol consumption [1,2].

Similar to the results of other studies, the frequency of death from MF after LTs in our study was 29% [37]. Patients with cirrhosis frequently experience MF, but the independent impact of the number or type of organ failures on LT outcomes is poorly understood [38]. Even in multiple organ failures, small retrospective studies have demonstrated that LT is feasible and has positive outcomes [38]. 

A previous study [11] observed that MF was the main cause of sudden death, with higher mortality during the first year after transplantation, followed by sepsis and GF, proving the vulnerability of AC patients in the hospital period after the transplant and outside. On the other hand, other studies corroborate the relationship between immunity and the cause of death [39,40].

According to a study, MF was the main factor in sudden death in AC patients receiving LTs, with higher mortality rates in the first year following transplantation. The results of this study also indicated that sepsis and GF were the primary killers [35]. However, additional research supports the link between immunity and the cause of death [39,40]. 

Leukocytes, monocytes/macrophages, and NK cells, which are the first line of the host’s defense and are specially equipped to survey their environment and respond swiftly to pathogen-derived threat signals through redistribution to the infection site in the body, are all cells of the innate immune system that express different receptors encoded by KIR genes. These cells serve as the first line of host defense [41,42,43].

In our study, the analysis of the frequency of KIR genes in the different causes of death showed that patients with alcoholic cirrhosis who died of multiple organ failure had a statistically significant increase in the frequency of the *KIR2DL2+* and *KIR2DS2+* genes. It should be noted that the increase in both genes corroborates the observations indicating that both genes are linked in our population [11,30], so it is impossible to know which of the two genes is involved in the development of multiple organ failure. Similar studies have found a statistically significant increase in the frequency of *KIR2DL2+* and *KIR2DS2+* genes in multiple organ damage patients with systemic lupus erythematosus [44] and systemic sclerosis [45].

In our study, the analysis of the relationship between the main causes of death and the frequency of KIR genes in patients with ascites and encephalopathy did not show statistically significant differences that could demonstrate an increase or decrease in susceptibility to a specific death cause, except for *KIR3DL1.* Patients without encephalopathy in AC showed increased GF frequency compared to patients with encephalopathy, corroborated by multivariate logistic regression analysis.

In this sense, *KIR3DL1* is a highly polymorphic receptor that binds to groups of HLA-A and HLA-B allotypes that express the Bw4 epitope. The difference in allotypes of *KIR3DL1* expresses itself at various stages. A typical allelic variant mainly encodes an activating rather than an inhibitory receptor (*KIR3DS1*) [46]. This fact could explain the results obtained in our study, which indicate that the allelic variants of this gene should be studied shortly.

Additionally, grade III/IV encephalopathy in patients with acute liver failure may progress to cerebral edema, a potentially fatal complication [47]. According to Bernal et al. [48], the prevalence of MF as a cause of death has increased, while clinical cerebral edema associated with acute liver failure has generally decreased. The incidence of cerebral edema may be much more common in areas where transplantation is less common.

On the other hand, analysis of how the KIR genes affected the causes of death in AC patients once again revealed that *KIR3DL1* impacted GF patient mortality. This influence could be seen both immediately and over time, and Cox’s regression multivariable analysis supported this. Three years after the transplant, it was also noted that the activator genes *KIR2DS1* and *KIR2DS4* impacted mortality. 

According to Legaz et al. [10], the presence of *KIR2DL3+* and *KIR2DS1+* in patients increased AR incidence when donor C2 ligands were present simultaneously. However, they did not examine how different causes of death affected the likelihood of survival. Everything thus points to how aKIRs can affect GF and patient mortality.

The main limitation of our study is the limited sample size of other causes of death (cardiac arrest, digestive bleeding, metastasis, edema lung, metastasis, pancreatitis, pneumonia, primary dysfunction, and viral relapse), which could not be analyzed individually in this study due to its small sample size that does not allow a deeper statistical analysis of association. However, we cannot discard potential influence in future similar studies with larger cohorts and/or extended analysis periods. Despite all these limitations, our data are statistically robust, with promising diagnostic implications achieved in this study.

## 5. Conclusions

In conclusion, our results show how the presence of the *KIR2DL2/S2+*, *KIR2DL5+*, and *KIR3DL1+* genes in the patient could increase the frequency of death from multiple organ failure or graft failure. Our findings demonstrate the AC patient’s susceptibility to LT inside and outside the hospital following the transplant. To improve and modify medical treatments for these patients to increase their short- and long-term survival, close medical monitoring and searching for biomarkers that indicate vulnerability to a specific cause of death are essential. In order to prevent death and enhance AC patient survival, it would be necessary to adhere to a protocol to avoid septic symptoms and GFs.

## Figures and Tables

**Table 1:** Baseline demographic, clinical, and biochemical characteristics of the male AC patients were analyzed in this study.

	Total AC Patients *N* = 164, *n* (%)
Recipient	
Mean age * (mean years ± SD)	54.3 ± 6.9
Sex (male/female)	150 (91.4)/14 (8.6)
Ascites+ Mean age	118 (71.9) 54.2 ± 1.3
Encephalopathy+ Mean age	39 (23.7) 56.7 ± 2.0
Causes of death	
Sepsis	39 (23.7)
Graft failure	32 (19.5)
Multiorgan failure	29 (17.7)
Others **	64 (39.1)

AC, alcoholic cirrhosis; *N*, total number of individuals; *n*, number of patients in each group; SD, standard deviation. * The mean values were analyzed (mean value ± SD) in all cases. ** Other causes of death include cardiac arrest, digestive bleeding, edema lung, metastasis, pancreatitis, pneumonia, primary dysfunction, and viral relapse.

**Table 2:** Frequency of KIR genes in AC patients with different causes of death.

	Causes of Death		
	Multiorgan Failure (MF) (*N* = 29)	Sepsis (S) (*N* = 39)	Graft Failure (GF) (*N* = 32)
KIR Genes	*n* (%)	*n* (%)	*n* (%)
**iKIRs**			
*KIR2DL1+/S1−*	19 (65.5)	23 (58.9)	22 (68.7)
*KIR2DL2+*	22 (75.8) ^a^	21 (53.8)	14 (43.7)
*KIR2DL3+*	26 (89.6)	36 (92.3)	30 (93.7)
*KIR3DL1+*	24 (82.7)	31 (79.4)	30 (93.7)
**aKIRs**			
*KIR2DS1+*	11 (37.9)	13 (33.3)	10 (31.2)
*KIR2DS2+*	22 (75.8) ^b^	20 (51.2) ^c^	14 (43.7)
*KIR2DS3+*	9 (31.0)	7 (17.9)	10 (31.2)
*KIR2DS4+*	24 (82.7)	31 (79.4)	30 (93.7)
*KIR2DS5+*	11 (37.9)	12 (30.7)	10 (31.2)

*N* is the total number of individuals; *n* is the number of individuals with a KIR gene. MF; Multiorgan failure; S; Sepsis, GF; Graft failure. All comparisons were made using the 2-tailed Fisher’s exact test. ^a^ OR = 0.247; 95% CI: 0.082–0.744, *p* = 0.018; *p* value obtained by comparing MF *KIR2DL2+* patients with GF *KIR2DL2+* patients. ^b^ OR =2.986; 95% CI: 1.037–8.594, *p* = 0.047; *p* value obtained by comparing MF *KIR2DS2+* patients with GF *KIR2DS2+* patients. ^c^ OR = 4.041; 95% CI: 1.344–12.146, *p* = 0.018; *p* value obtained by comparing MF *KIR2DS2+* patients with GF *KIR2DS2+* patients.

**Table 3:** Logistic regression multivariable analysis for main causes of death.

	Wald	*p*	OR	95% CI	
				Lower	Upper
**Sepsis**					
*KIR2DL2*	0.785	0.376	0.432	0.067	2.768
*KIR2DL5*	0.000	0.996	1.007	0.064	15.856
*KIR3DL1*	4.056	**0.044**	0.025	0.001	0.906
*KIR2DS1*	0.989	0.320	0.176	0.006	5.413
Ascites	1.371	0.242	3.432	0.436	27.042
Encephalopathy	0.148	0.700	0.698	0.112	4.364
**Graft failure**					
*KIR2DL2*	1.580	0.209	0.316	0.052	1.907
*KIR2DL5*	0.141	0.707	1.636	0.126	21.272
*KIR3DL1*	0.117	0.732	1.812	0.060	54.295
*KIR2DS1*	0.064	0.801	1.468	0.075	28.890
Ascites	2.517	0.113	0.117	0.008	1.658
Encephalopathy	1.068	0.301	2.825	0.394	20.245
**Multiorgan failure**					
*KIR2DL2*	3.148	0.076	8.911	0.795	99.821
*KIR2DL5*	2.652	0.103	0.122	0.010	1.535
*KIR3DL1*	1.008	0.315	0.144	0.003	6.333
Ascites	0.190	0.663	1.621	0.184	14.241
Encephalopathy	1.359	0.224	0.270	0.030	2.443

OR, an odds ratio with a confidence interval (CI) of 95%. *p*-values marked in bold are statistically significant (*p* < 0.05).

**Table 4:** Cox regression multivariable analysis for AC patients’ survival.

	Wald	*p*	OR	95% CI	
				Lower	Upper
**Age**	0.719	0.396	2.033	0.394	10.489
***KIR3DL1***	3.443	**0.034**	3.979	0.925	17.110
***KIR2DS1***	3.520	**0.041**	2.736	0.956	7.831
***KIR2DS3***	0.088	**0.026**	1.140	0.479	2.715
***KIR2DS4***	0.064	0.801	1.468	0.075	18.890

OR. odds ratio with a confidence interval (CI) of 95%. *p*-values marked in bold are statistically significant at *p* < 0.1.

## Data Availability

Data is unavailable due to privacy or ethical restrictions.
